# Chemotherapeutic agent 5-fluorouracil increases survival of SOD1 mouse model of ALS

**DOI:** 10.1371/journal.pone.0210752

**Published:** 2019-01-14

**Authors:** Amaya Rando, Miriam de la Torre, Anna Martinez-Muriana, Pilar Zaragoza, Antonio Musaro, Sara Hernández, Xavier Navarro, Janne M. Toivonen, Rosario Osta

**Affiliations:** 1 LAGENBIO, Departamento de Anatomía, Embriología y Genética Animal, Facultad de Veterinaria, Universidad de Zaragoza, Zaragoza, Spain; 2 Instituto Agroalimentario de Aragón-IA2 (Universidad de Zaragoza-CITA), Zaragoza, Spain; 3 Instituto de Investigación Sanitaria Aragón, Centro de Investigación Biomédica de Aragón (CIBA), Zaragoza, Spain; 4 Institute of Neurosciences and Department of Cell Biology, Physiology, and Immunology, Universitat Autònoma de Barcelona, Bellaterra, Spain; 5 Centro de Investigación Biomédica en Red sobre Enfermedades Neurodegenerativas (CIBERNED), Bellaterra, Spain; 6 DAHFMO-Unit of Histology and Medical Embryology, Sapienza University of Rome, Laboratory Affiliated to Istituto Pasteur Italia—Fondazione Cenci Bolognetti, Rome, Italy; 7 Center for Life Nano Science@Sapienza, Istituto Italiano di Tecnologia, Rome, Italy; 8 Departament de Medicina Experimental, Grup Patologia Neuromuscular Experimental, Facultat de Medicina, Universitat de Lleida/IRBLLEIDA, Lleida, Catalonia, Spain; Boston University School of Medicine, UNITED STATES

## Abstract

Amyotrophic lateral sclerosis (ALS) is a lethal motor neuron disease with no cure. Currently there are only two ALS drugs approved by the FDA, both with a limited therapeutic effect. In the search for drug candidates for ALS, we studied the effect of known stem cell mobilizing agents (treatment) and antimetabolite 5-fluorouracil (5-FU) (anti-treatment) in SOD1G93A model of ALS. Surprisingly, we found that anti-cancer drug 5-FU increases lifespan, delays the disease onset and improves motor performance in ALS mice. Although we were not able to demonstrate the mechanistic basis of the beneficial 5-FU action in ALS mice, our findings suggest that 5-FU or similar drugs are possible drug candidates for the treatment of motor neuron diseases through drug repurposing.

## Introduction

Amyotrophic lateral sclerosis (ALS) is a devastating disease characterized by upper and lower motor neuron (MN) degeneration, progressive muscle paralysis and atrophy [1]. ALS patients typically die from cardiorespiratory failure within 2–5 years after the diagnosis. The only FDA accepted treatments for ALS, riluzole and edaravone, appear to provide only limited benefit to the patients. Thus, new treatments are desperately needed for fighting ALS [2]. While new drugs, cell-based therapies and gene targeting are being investigated for clinical use, the progress is slow because of bottlenecks in the therapeutic development process. Drug repurposing (using an agent already commercialized to treat one disease for the treatment of other diseases) is one of the strategies to reduce the time required to get new treatments on the market. Because repurposing is built on previous R&D efforts, their review by the regulatory agencies is accelerated and new candidate therapies may reach clinical trials and integrate into the clinic more quickly. Several well-known drugs commercialized for other diseases are being investigated in the search for ALS treatment and some of them have already reached clinical trials [3].

In the search for drug candidates for ALS treatment, we have recently studied stem cell mobilizing agents, some of which have been shown to positively affect the outcome in the SOD1G93A model of ALS [4–7]. As an additional “control” treatment that we reasoned to have an opposite (i.e. temporary negative) effect on circulating hematopoietic stem cells and white blood cell (WBC) count, we used low dose of the well characterized, FDA-approved anti-cancer drug 5-fluorouracil (5-FU). The principal known action of 5-FU is inhibition of DNA synthesis by blocking the activity of thymidylate synthase (TYMS, EC 2.1.1.45) which causes an imbalance of the nitrogen bases normally used for DNA synthesis and misincorporation of uracil into DNA [8]. Although less studied, 5-FU can also induce striking alterations in RNA metabolism, such as impairments in messenger RNA (mRNA) and ribosomal RNA (rRNA) synthesis, splicing and post-transcriptional modification [9, 10]. Because 5-FU toxicity is related with cell division, cells with higher replicative rate (such as cancer cells, hematopoietic cells and intestinal enterocytes) are more sensitive to the drug.

As expected, we observed a temporary decrease in WBC and hematopoietic stem cells after 5-FU administration which was fully recovered within two weeks of the treatment. Against our initial hypothesis, we also found that, instead of aggravating the disease in SOD1G93A mice, 5-FU increased the lifespan of the treated animals, delayed the disease onset and improved the motor performance measured by rotarod test. Although 5-FU did not significantly modulate motor neuron survival, reactive gliosis or muscle morphology our results suggest that a low-dose regimen of 5-FU or its analogs may have beneficial effects on ALS and encourage further pharmacological and mechanistic studies for their use as repurposed ALS drugs.

## Material and methods

### Animals

All procedures were approved by the Ethic Committee for Animal Experimentation of the University of Zaragoza (permisson number PI18/17). Animals were taken care according to the Spanish Policy for Animal Protection RD53/2013 and the EU Directive 2010/63. The transgenic mice B6SJLTg(SOD1G93A)1Gur/J [11] expressing a high copy number of the G93A mutant form of human SOD1 were purchased from The Jackson Laboratory and housed in the animal facilities of the UZ. Food and water were available ad libitum. SOD1G93A colony was maintained by breeding hemizygous SOD1G93A males with B6SJL wild-type females. The genotyping of the offspring was performed as described in the in the Jackson Laboratory protocol. The animals were maintained in conditions of environmental enrichment (such as paper rolls) and were examined daily to avoid suffering. The humane endpoint for experimental animals was established as the moment the mice were unable to right themselves within 30 seconds after being placed on their side and the age at this moment was considered as death for survival analysis [12]. Animals were euthanized individually with CO_2_.

### Administration of 5-FU

To determine the effect of 5-FU on blood cell counts, ten SOD1G93A mice at age of 10 weeks were subjected to a single intraperitoneal injection of 5-FU at 150 mg/kg. The drug was freshly diluted in saline (15 mg/ml) and the volume of each individual injection was between 0.18 and 0.32 ml. For the behavioural tests, survival assay and serial blood extractions to determine the evolution in the number of hematopoietic stem cells (see below), SOD1G93A mice were injected as with 5-FU or with saline every two weeks (age of 10, 12 and 14 weeks). This fortnightly dose corresponds to a low dose of 12.2 mg/kg, or 450 mg/m^2^, in humans [13]. The total number of mice used for survival assay was 58 (29 treated with 5-FU and 29 with saline), of which 22 were also used for behavioural assays (11 treated with 5-FU and 11 with saline). Serial blood sampling was conducted on 20 mice (10 treated with 5-FU and 10 with saline).

### Cell counts in peripheral blood and the bone marrow

Blood and bone marrow were collected before the treatment (t = 0, n = 4) and at days 4 (n = 4), 14 (n = 4) and 20 (n = 2) after the single 5-FU injection and compared with basal conditions (t = 0). Briefly, blood was extracted by cardiac puncture and 100 μl of total blood cells were quantified (cells per ml) by ABACUS Junior Vet Hematology Analyzer (Diatron). From each mouse, the bone marrow from both femurs was obtained by flushing the medullary cavity with phosphate buffered saline (PBS). Suspended bone marrow cells were stained with Quick Panoptic kit (Analytix), centrifuged 400 x g for 5 min and resuspended in 1% bovine serum albumin (BSA) in PBS. After treatment with erythrocyte lysis buffer (Sigma-Aldrich) samples were centrifuged as above and the bone marrow mononuclear cells (BMMC) were counted and expressed as BMMC per femur.

### Serial blood extractions and hematopoietic stem cell determination

Before the first 5-FU administration (day 65) and five days after every injection (days 75, 90 and 105), blood samples were extracted from the tail vein to analyze the effect of 5-FU administration on circulating hematopoietic stem cells. Blood samples were collected and prepared for immunostaining as previously described [7] and incubated for 30 minutes with following antibodies: anti-mouse Ly-6A/E (Sca-1) PE (12–5981 eBioscience), anti-mouse CD117 (c-Kit) APC (17–1171 eBioscience), mouse hematopoietic lineage eFluor 450 Cocktail (eBioscience 88–7772) and PE-Cy7 rat anti-mouse CD127 (560733 BD Biosciences). The number of hematopoietic stem cells (HSCs; lin-, Sca-1 +, c-kit +), common myeloid progenitors (CMPs; lin-, Sca-1 -, c-kit +), and common lymphoid progenitors (CLPs; Lin-, CD127+) were determined using a Gallios flow cytometer (Beckman Coulter) and the output was analyzed with Kaluza software (Beckman Coulter). The data were expressed as a percentage of the total selected events.

### Behavioral tests

Two sex balanced groups of SOD1G93A mice (n = 11 per group) were randomly distributed to evaluate the progression of the disease and locomotor function by rotarod and hang-wire tests. Starting at the age of ten weeks (before the first injection), these tests were performed weekly until the mice reached the endpoint criteria following the international guidelines for preclinical studies with ALS mice [14]. In the rotarod test, the time mice were able to maintain their balance on the wheel was recorded. For assessing muscle strength, the mice were placed on a wire lid, gently turned upside down and the latency to fall was timed. In both tests, animals were given three opportunities to reach a maximum of 180 seconds and only the best performance was considered. Weight of each animal was recorded weekly and the onset of the disease for each animal was assed at the moment the particular mouse did not increase in weight any more.

### Gene expression

At the age of 15 weeks (five days after the last injection), plasma and skeletal muscle from 5-FU treated or vehicle treated SOD1G93A mice, as well as wild-type littermates (n = 8 for all groups), were collected and processed for gene expression analysis. Left *quadriceps femoris* muscles were dissected and immediately frozen in liquid nitrogen. Each muscle was pulverized in liquid nitrogen using a Cellcrusher cryogenic tissue pulverizer (Cellcrusher, Cork, Ireland) and half of the power was kept at -80°C for protein extraction (below). The powered muscle tissue was further homogenized using a PRO200 homogenizer (PRO Scientific Inc) and RNA was extracted with TRIzol reagent (Invitrogen). Potential residual genomic DNA was eliminated using the Turbo DNA-free Kit (Ambion) and 1μg of DNAse-treated RNA was retrotranscripted using the Superscript First Strand kit (Invitrogen). Quantitative real-time PCR (qRT-PCR) was performed from 1:10 diluted cDNA in triplicates using StepOne Plus Real-Time PCR System (Applied Biosystems). The gene-specific TaqMan probes (Applied Biosystems) used are indicated in Table 1. Geometric mean of the reference genes Gapdh and Actb (β-actin) was used for normalization [15] and relative gene expression was determined using the 2^-ΔΔCT^ method [16].

**Table 1:** Taqman gene expression assays used in the study.

Gene Symbol	Part Number
Ankrd1	Mm00496512_m1
Col19a1	Mm00483576_m1
Gsr	Mm00833903_m1
Mt2	Mm00809556_s1
Myog	Mm00446194_m1
Snx10	Mm00511049_m1
Bax	Mm00432050_m1
Bcl2	Mm00477631_m1
Casp1	Mm00438023_m1
Casp3	Mm01195085_m
Atg5	Mm00504340_m1
Becn1	Mm00517174_m1
E2f1	Mm00432939_m1
Map1lc3a	Mm00458724_m1
p62/Sqstm1	Mm00448091_m1
Actb	4352933E
Gapdh	4352932E

### Protein expression

Powdered tissue (see above) was homogenized in RIPA lysis buffer containing protease inhibitors (Roche). The homogenate was centrifuged at 10000 ×g for 10 min at 4°C, the supernatant was collected and the protein concentration was determined by BCA method (Sigma Aldrich). Forty micrograms of total protein were subjected to SDS/PAGE and transferred to PVDF membranes (Amersham Biosciences). For immunodetection, membranes were blocked overnight in 5% skimmed milk at 4°C and then incubated one hour with the primary antibody anti-hSOD1 (HPA001401 Sigma-Aldrich). After washes, the membranes were incubated with HRP-conjugated anti-rabbit secondary antibody (sc-2004 Santa Cruz 1:3000), washed again and finally incubated with enhanced chemiluminescent reagent (GE Healthcare Life Science). Immunoblots were exposed and scanned, and quantitative densitometry was performed with AlphaEaseFC software (Bonsai).

### Electrophysiological tests

SOD1G93A mice were injected with 5-FU (n = 6) or saline (n = 6) as indicated above and motor nerve conduction tests were performed before the first injection (at the age of 10 weeks) and at the age of 12 (one injection), 14 (two injections) and 16 (three injections) weeks. The sciatic nerve was stimulated and the compound muscle action potential (CMAP) from tibialis anterior and plantar interossei miscles was recorded as previously described [17].

### Histological and immunohistochemical processing

After the last electrophysiological tests at the age of 16 weeks, mice were transcardially perfused with 4% paraformaldehyde in PBS (Gibco) and the lumbar segment of the spinal cord was removed. After post-fixation and cryopreservation, transverse sections (40 μm) were serially cut with a cryotome (CM190, Leica Microsystems) between L3 and L5 segmental levels. MN count was performed as previously described [18]. For immunohistochemistry, sections were blocked with PBS-triton fetal bovine serum (Sigma Aldrich) and incubated overnight with primary antibodies for anti-glial fibrillary acidic protein (GFAP 1:1000, Dako) or rabbit anti-ionized calcium binding adaptor molecule 1 (Iba11:1000, Wako). After washing with PBS, sections were incubated with Alexa Fluor 488- or 594-conjugated secondary antibody for one hour at room temperature (1:200, Jackson Immunoresearch). Astroglial and microglial inmunoreactivity quantification was performed as previously described [17].

### Statistical analysis

Statistical analysis for behavioral assays, electrophysiology tests, cell proliferation and gene expression was performed by means of ANOVA or Student´s t-test. Survival over time was computed using Kaplan-Meier estimates and the survival distributions (as well as disease onset) of treated vs non-treated animals were tested with Mantel-Cox log-rank test. Values were considered statistically significant (*) at P<0.05. Tendency (^) towards significance at P<0.1 is indicated in some cases.

## Results

### The effect of 5-FU administration on peripheral blood and bone marrow

**Fig 1:**
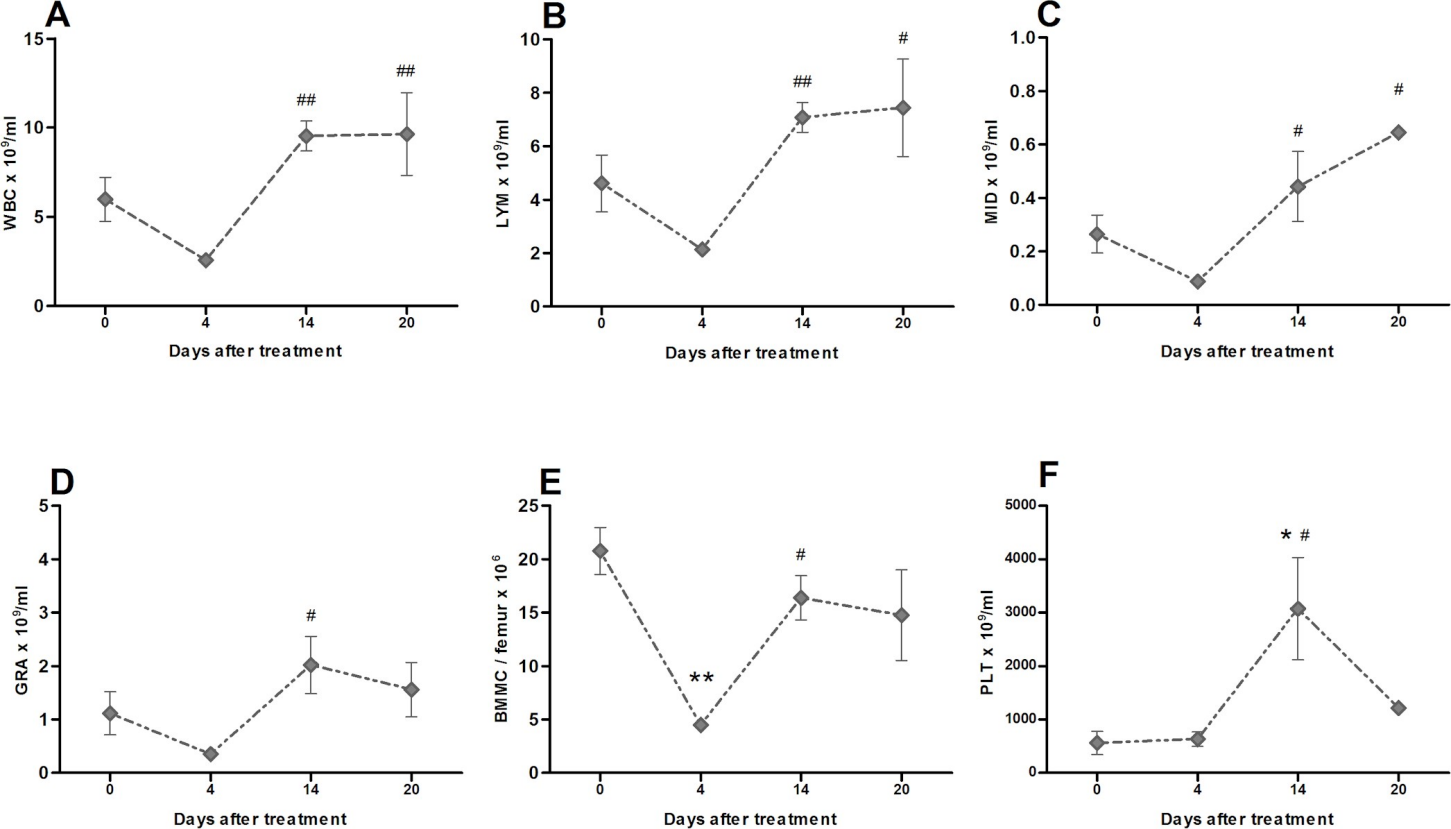
Long-term hematologic alterations in SOD1G93A mice in response to single 5-FU administration. Blood samples were analyzed before 5-FU treatment (0 days) and up to 20 days after 5-FU administration. (A) Total WBC, (B) lymphocytes, (C) monocytes (mid-range absolute count, MID) and (D) granulocytes are expressed as number of cells x10^9^ per ml of blood. (F) Bone marrow cellularity expressed BMMC x 10^6^ per femur and (E) platelets (PLT) as number of cells x10^9^ per ml of blood. Data is shown as mean (n = 4, except day 20 n = 2) +/- SEM. The asterisk (*) and the hash (#) denote significance compared with pre-treatment and with t = 4, respectively. One way ANOVA with Tukey’s test, */# p<0.05, **/## p< 0.01.

Granulocyte colony stimulating factor (GCSF) and its analog Pegfilgrastim increase the mobilization of hematopoietic stem cells and blood WBC count [6, 7]. Initially, our aim was to use 5-FU as an opposite treatment compared to GCSF. To confirm the expected action of 5-FU, we measured the effect of 5-FU administration on the number of the circulating white blood cells (WBC) in the SOD1G93A mice following the treatment with a single dose of 5-FU at 150 mg/kg. Hemograms were performed at 4, 14 and 20 days after 5-FU administration (t = 4, t = 14, t = 20) and compared with baseline counts before the treatment (t = 0) (Fig 1). At t = 0, all the measured parameters were within the expected physiological values. In agreement with earlier studies that used the same dosage and delivery route [19], we obtained the minimum values for total WBCs (Fig 1A), lymphocytes (Lym, Fig 1B), monocytes (MID, Fig 1C) and granulocytes (Gra, Fig 1D) four days after 5-FU administration. This reduction was rapidly recovered by the proliferative capacity of the bone marrow since the levels of circulating WBC (Fig 1A) and BMMC of the bone marrow (Fig 1E) reached physiological values at day 14 and 20. The number of red blood cells, hemoglobin and the hematocrit maintained within physiological levels and were not affected by 5-FU (S1 Fig). However, in agreement with previous studies [20], 5-FU administration promoted a strong temporal increase in the number of platelets 14 days after treatment (Fig 1F).

**Fig 2:**
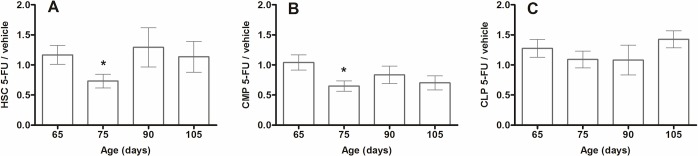
Circulating hematopoietic stem cells in SOD1G93A mice after fortnightly 5-FU administration. Data is shown as the mean fold change +/- SEM in the percentage of (A) HSC, (B) CMP and (C) CLP in 5-FU treated SOD1G93A mice compared with the mean of vehicle-treated SOD1G93A mice (n = 10 per group). Day 65 are the pre-treatment samples and days 75, 90 and 105 are each taken after 4 days from last 5-FU administration. *p<0.05.

Homeostasis of the hematopoietic system can be maintained by a rapid response to the loss of progenitors to restore the steady-state. Using flow cytometry analysis, a rapid decline in circulating hematopoietic stem cells (HSC, Fig 2A) and common myeloid precursors (CMP, Fig 2B) was observed. Similar, although statistically insignificant pattern was observed in common lymphoid precursors (CLP, Fig 2C). However, in two weeks following an intraperitoneal 5-FU administration at 150 mg/kg, the levels of circulating and bone marrow WBC, as well as circulating HSC were restored to basal levels. These results prompted us to investigate a treatment regimen for the behavioural and lifespan studies, in which 5-FU at 150 mg/kg was delivered once every two weeks.

### Low-dose 5-FU administration prolongs life span and improves locomotor function in SOD1G93A mice

**Fig 3:**
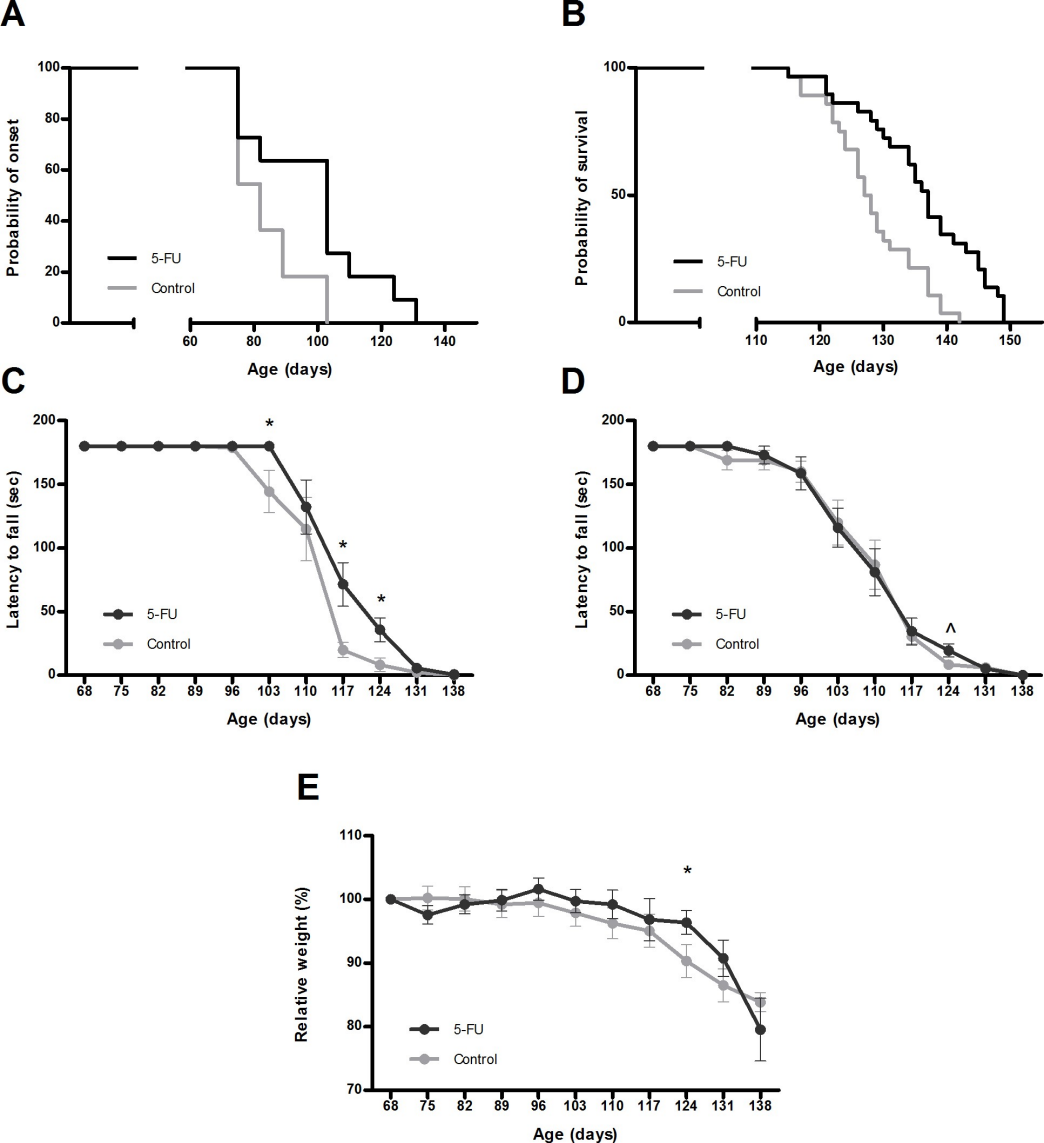
5-FU increases lifespan, improves rotarod performance and mitigates weight loss of SOD1G93A mice. SOD1G93A mice were treated with 5-FU every two weeks starting at age of 70 days. In each graph, groups of 5-FU treated (dark grey) and control (light grey) SOD1G93A mice are shown. (A) Cumulative probability of the onset of disease, controls 82 days and 5-FU treated 117 days (log-rank test p = 0.027). (B) Cumulative probability of survival, controls 127.5 days and 5-FU treated 137 days (log-rank test p = 0.0003). Latency to falling when submitted to the (C) rotarod or (D) hang-wire test. (E) Relative weight evolution during the disease progression. * p< 0.05; ^p<0.1 (tendency). Note that the X-axes for the panels A and B are cut for clarity.

SOD1G93A mice have a shortened lifespan and show progressive locomotor defects during the symptomatic stage of the disease. 5-FU administration was initiated at the age of 70 days, a late presymptomatic stage in which SOD1G93A mice do not yet show overly clinical features (such as weight loss or impaired locomotory function) but electrophysiological abnormalities can be detected [17]. It was found that fortnightly intraperitoneal 5-FU injections at 150 mg/kg delayed the median onset of the disease (controls 82 days and 5-FU treated 117 days, log-rank test p<0.01) as measured by initiation of the weight loss (Fig 3A). In the same line, median lifespan of 5-FU treated mice was significantly prolonged (controls 127 days and 5-FU treated 137 days, log-rank test p<0.001) (Fig 3B). The effects of 5-FU administration on motor coordination and grip strength were tested by means of rotarod and hang wire tests, respectively. On the rotarod (Fig 3C), vehicle control SOD1G93A mice started to decline in performance at the age of 103 days, whereas all 5-FU treated mice were still able to properly perform the test (p<0.05). From this age, 5-FU treated mice performed significantly better on the rotarod at 117 and 124 days (p<0.05) compared to untreated littermates, showing a delay in the progression of the disease. Muscle strength, assessed by hang-wire test, was not significantly affected although a close to significant positive effect of 5-FU was observed at late stage of the disease (age of 124 days, p = 0.06) (Fig 3D). Body weight was recorded weekly (Fig 3E). Besides a slight (non-significant) weight loss after the first administration, 5-FU treated mice recovered the following week and exhibited generally milder mutant SOD1-associated weight loss in the symptomatic stage compared to untreated mice. From 117 to 131 days, the vehicle control group lost more than 10% of body weight whereas 5-FU -treated group conserved their initial weight until 124 days (p<0.05). Together, the above data indicates that 5-FU exerts a therapeutic effect on the progression of the disease as it prolongs life span, improves the locomotor behavior and delays terminal weight loss of the ALS mice.

### 5-FU does not protect motor neurons or reduce reactive gliosis in SOD1G93A mice

**Fig 4:**
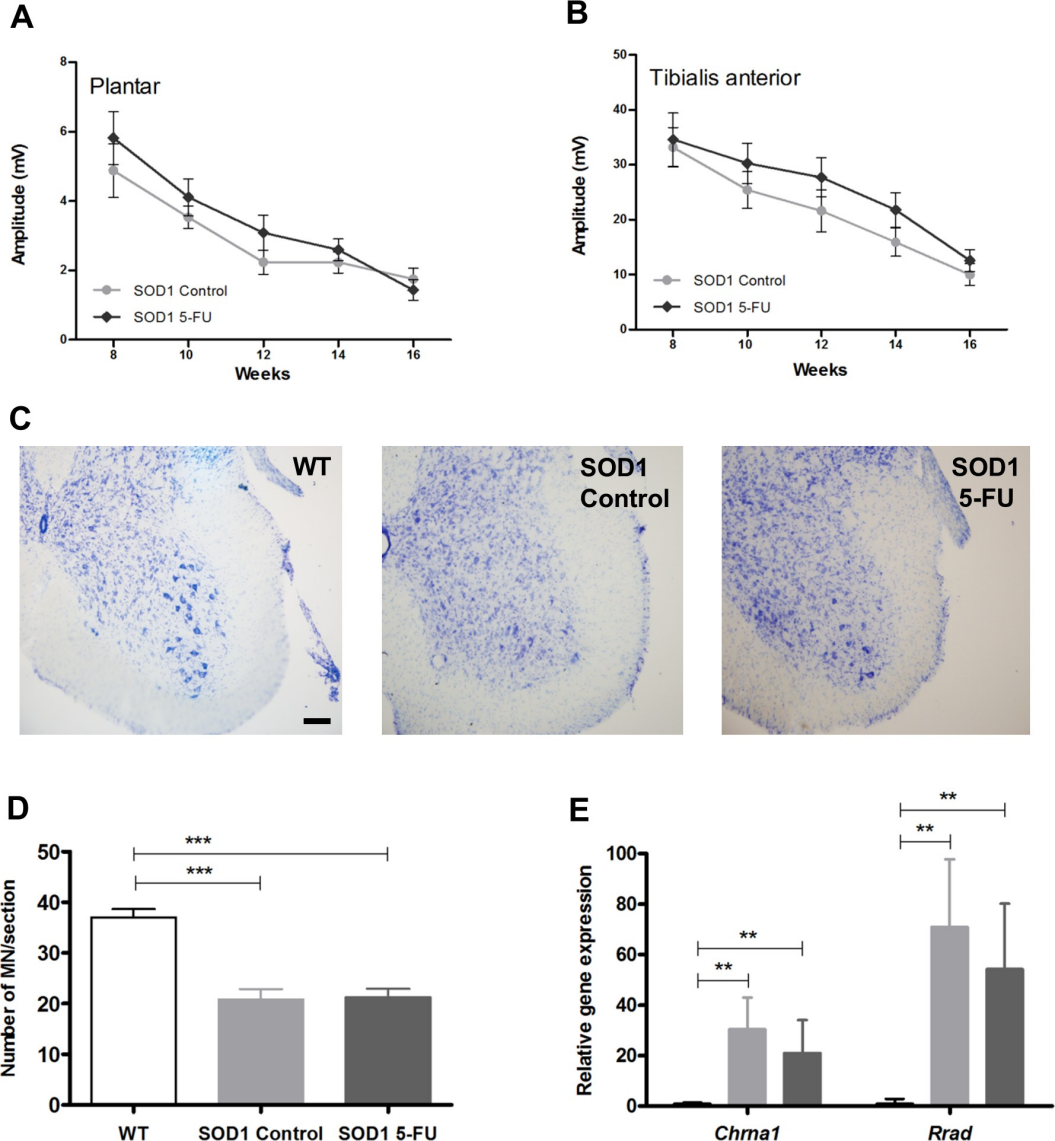
Electrophysiology and motor neuron (MN) survival. Lower motor function was evaluated in (A) plantar and (B) tibialis anterior (TA) muscles at 8–16 weeks of age in non-treated (control, light grey) and 5-FU treated (5-FU, dark grey) SOD1G93A mice by measuring the amplitude of the CMAP. Values are means (n = 6 per group) +/- SEM. (C) Representative images of Nissl stained L4 spinal cord sections from wild-type (WT), non-treated (SOD1 Control) and 5-FU treated (SOD1 5-FU) SOD1G93A mice at age of 16 weeks. Scale bar 100 μm. (D) Number of MN counted per spinal cord section in WT (white bars), non-treated (SOD1 control, light grey bars) and 5-FU (SOD1 5-FU, dark grey bars) SOD1G93A mice. Data are shown as mean of sections counted from each mouse (n = 7 for wt, 10 per group for SOD1G93A) ± SEM. (E) Relative quantification of Chrna1 and Rrad transcript levels in wild-type (WT), non-treated (control) and 5-FU treated (5-FU) SOD1G93A mice at age of 15 weeks. One way ANOVA with Tukey’s test, ** p<0.001, *** p<0.0001.

Distal axonal degeneration initiates early during the disease, before the death of MN cell bodies, in ALS patients and in animal models of the disease [21]. Lower MN functional state was assessed by motor nerve conduction tests between 8 and 16 weeks of age. No significant differences were found in the amplitude of CMAPs in 5-FU treated vs. vehicle group in plantar (Fig 4A) or tibialis anterior (TA, Fig 4B) muscles although 5-FU administration had a marginal effect on TA CMAP from 10 to 14 weeks. MN counts in the lateral ventral horns of lumbar spinal cord sections revealed almost 50% loss in SOD1G93A mice compared with their wild-type littermates at 16 weeks of age (Fig 4C). 5-FU treatment did not improve sparing of MNs as they were lost equally in both treated and non-treated SOD1G93A mice (Fig 4D). *Chrna1* (cholinergic receptor, nicotinic, alpha 1) and *Rrad* (Ras-related associated with diabetes), genes whose expression levels are upregulated by denervation and in SOD1G93A animals [22–25], were not significantly affected by 5-FU (Fig 4D).

**Fig 5:**
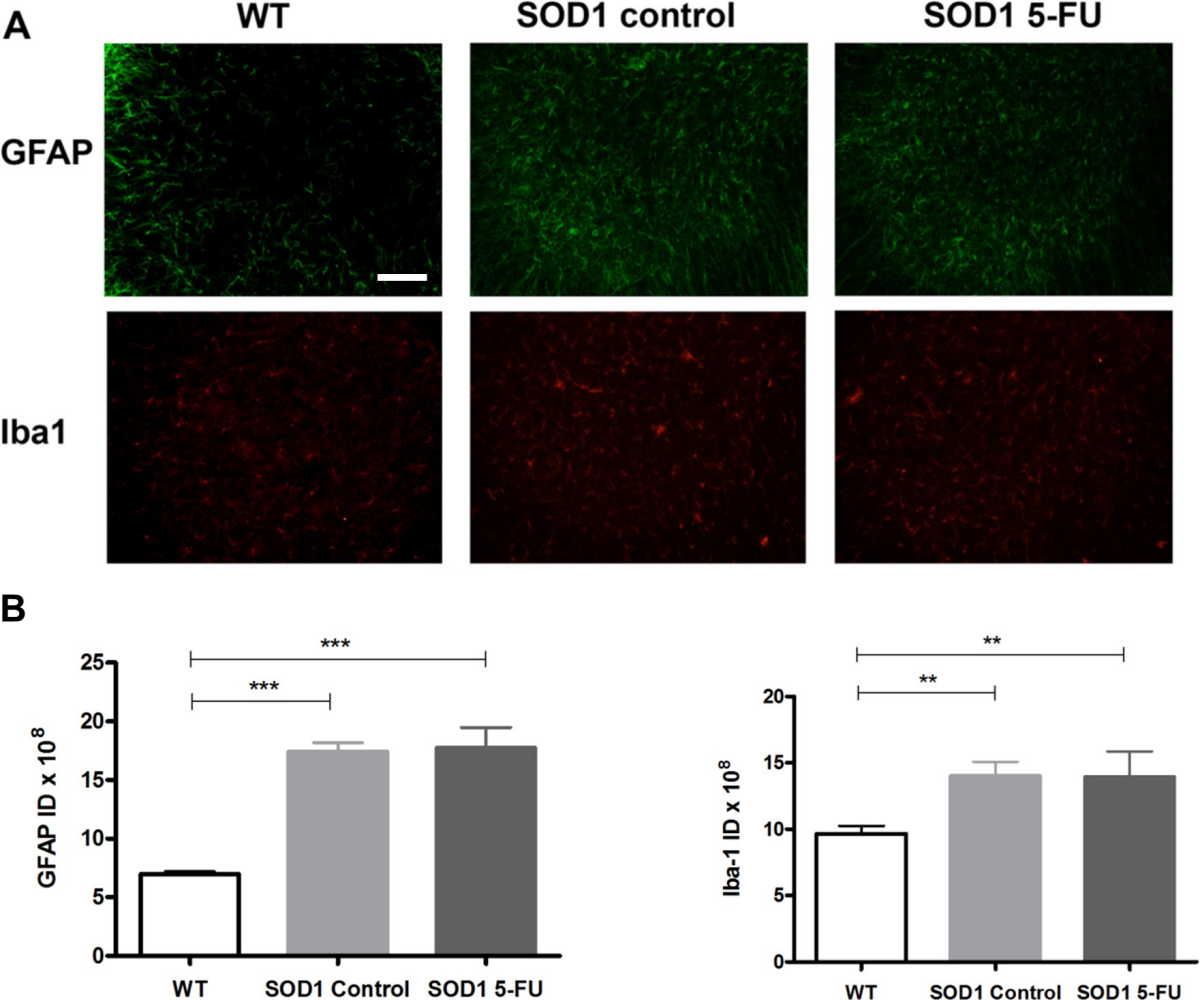
Analysis of glial reactivity in SOD1G93A mice. (A) Representative microphotographs of spinal cord ventral horns from wild type, control SOD1G93A and 5FU treated SOD1G93A mice immunolabelled with markers for astrocytes (GFAP) and microglia (Iba1). Scale bar 125 μm. (B) Histograms representing the quantification of GFAP and Iba1 immunoreactivity in vehicle control (SOD1 control, light grey bars) and 5-FU treated (SOD1 5-FU, dark grey bars) SOD1G93A mice compared to wild-type (WT, white bars) mice. The integrated density (ID) is the area above the threshold for the mean density minus the background. One way ANOVA with Tukey’s test, ** p<0.001, *** p<0.0001.

Next, immunofluorescence was performed against GFAP and Iba1, markers for astrocytes and microglia, respectively, to analyze reactive glial response in the spinal cord. SOD1G93A mice from both treated and non-treated groups showed an evident increase of astrocytosis and microgliosis (Fig 5). Quantification of the immunoreactivity demonstrated that 5-FU treatment did not modulate the reactive gliosis in SOD1G93A mice. The above data suggests that 5-FU does neither lead to spinal MN protection nor reduce reactive gliosis, and its possible therapeutic effect should be based on other mechanisms of action.

### 5-FU does not ameliorate commonly elevated SOD1G9A muscle markers

**Fig 6:**
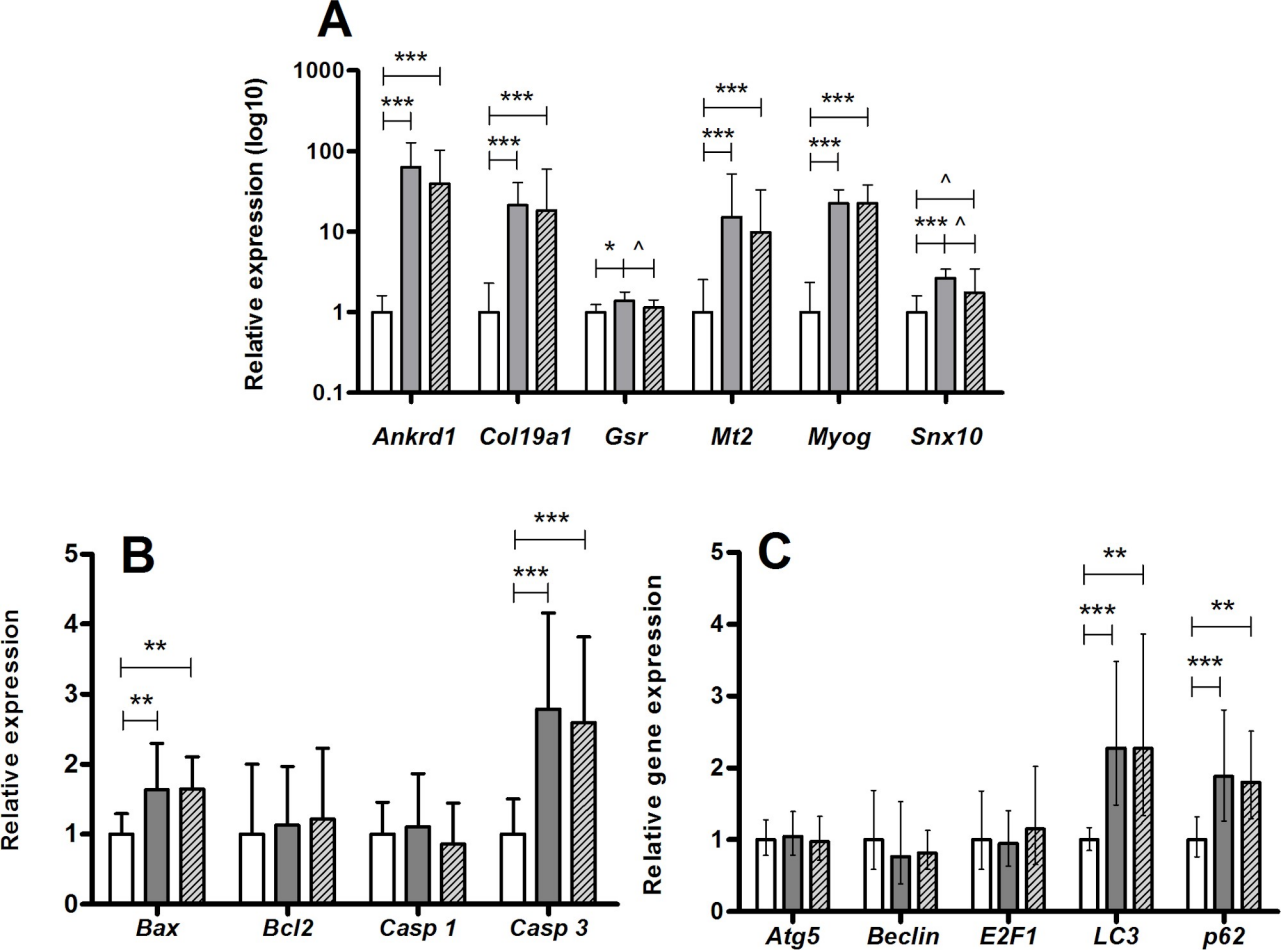
Gene expression profile in skeletal muscle. The graphics show the fold change in transcript levels in non-treated SOD1G93A muscles (gray bars) and 5-FU treated SOD1G93A muscles (dashed bars) compared with their respective wild-type littermates (white bars) at 105 days of age. (A) Fold change in the transcript levels of Ankrd1, Col19a1, Gsr, Mt2, Myog and SNx10. (B) Fold change in the expression of the apoptotic regulators Bax,Blc2, Casp1 and Casp3. (C) Transcript levels of the autophagy mediator´s genes Atg5, Beclin, E2F1, LC3, and p62. Data are shown as mean values ± SEM.*p <0.05; **p <0.001; ^ p <0.1 (tendency).

To search for molecular evidence, we analyzed the expression of genes involved in muscle homeostasis and metabolism that were previously shown to be induced in the SOD1G93A mice [23, 24]. As expected, in symptomatic mutant mice at 15 weeks of age, the expression of *Ankrd1*, *Col19a1*, *GSr*, *Mt2*, *Myog* and *Snx10* were upregulated compared to their wild-type littermates (Fig 6A). However, there were no differences observed between 5-FU treated and control SOD1G93A animals besides a tendency for reduced expression of *Gsr* (*glutathione reductase*) and *Snx10* (*sortin nexin 10*) (p<0.1). Equally, apoptotic markers *Bax* (*BCL2-associated X protein*) and *Casp3 (Caspase 3)*, as well as autophagy markers *Lc3* (*Map1lc3a*) and *p62* (*Sqstm1*), were all upregulated in both untreated and 5-FU-treated SOD1G93A muscles compared with wild type (Fig 6B and 6C). Hence, the above data suggests that, at least at transcript level, there is no major effect by 5-FU on myogenic, apoptotic or autophagic markers commonly elevated in SOD1G9A muscles.

## Discussion

The results of the present study indicate that treatment with a low dose of 5-FU increases the lifespan, delays the disease onset and slightly improves the motor performance of the SOD1G93A mice. Although we were not able to demonstrate the cellular targets behind the beneficial effect of 5-FU, we shall next discuss the potential importance of the findings suggest further studies in order to address the mechanistic basis of the drug effect in the ALS model.

Analogs of granulocyte colony stimulating factor (GCSF) are used in clinic to treat neutropenia in patients receiving myelosuppressive drugs. GCSF stimulates hematopoietic precursors of the bone marrow as well as hematopoietc stem cell (HSC) mobilization to the bloodstream. Long term treatment with GCSF analog pegfilgrastim attenuates reactive gliosis and increases survival of SOD1G93A mice [6], possibly increasing turnover of microglia and infiltration of monocytes into peripheral tissues to modulate ALS-associated inflammatory response. Here, the rationale for testing 5-FU in a mouse model of ALS was to investigate an agent that has an opposite effect on peripheral WBC and HSC. A single dose of 5-FU eliminates the majority of the hematopoietic system, i.e. cells that are actively dividing. However, quiescent, most primitive stem cells are saved, triggered to cycle and reconstitute the whole hematopoietic system within 2 weeks of 5-FU administration [26, 27]. This is in line with our data since we found that peripheral and bone marrow WBC were completely recovered in two weeks, when the next dose of 5-FU was administered.

Although the short-term effects of 5-FU on circulating WBC and HSC was negative, the long-term treatment may also induce beneficial effects. In a study on HSC ageing, [28] 12-week-old mice were injected with 5-FU using a similar treatment regime (150 mg/kg IP, once in three weeks). After the initial drop, the long term assessment revealed that HSC subset was increased after the last 5-FU treatment (2 to 4 treatments). However, these cells were functionally challenged due epigenetic alterations, apparently caused by an increased number of cell duplications [28]. Thus, forcing the quiescent stem cells to replicate with low-dose 5-FU pulses may have beneficial effects. However, the possibility that the treatment promotes ageing of the stem cell populations needs to be considered. For this reason, it would be important to first determine the minimal effective concentration of 5-FU. Secondly, combined treatment strategies or related but less toxic substances should be investigated. These could include combined treatments with agents that optimize TS binding of 5-FU (leucovorin), enhance 5-FU bioavailability and activation (Uracil/Ftorafur, Methotrexate) or 5-FU prodrugs that allow alternative delivery route and side-effect minimization (Capecitabine, Tegafur).

Most 5-FU is metabolized, mainly in the liver, to 5-dihydrofluorouracil (DHFU) which in turn, after further metabolic conversions, is secreted as 2-fluoro-beta-alanine (FBAL) through the kidney. However, to be bioactive, i.e. to kill fast-replicating cells, 5-FU needs to be metabolized via other route to fluorouridine monophosphate (FUMP) that can be further converted to its active metabolites responsible for TS inhibition, DNA and RNA damage. It is possible that some of the cellular consequences of 5-FU metabolites important for beating cancer cells are also involved in the improvement of the phenotypic outcome in SOD1G93A mice. Cell cycle inhibitors such as 5-FU block cell proliferation at high concentrations. At low concentrations, however, they have been shown to *promote* proliferation of some cancer cells in vitro [29]. This type of “hormetic dose–response” may be caused by a direct stimulation or after an initial homeostatic disruption followed by an overcompensation response. Besides, stress at low levels may elicit adaptive beneficial responses that may protect against subsequent exposure to severe stress [30]. Oxidative stress is thought to play a major role in ALS pathogenesis [31, 32]. Moreover, 5-FU is known for inducing oxidative stress in bone marrow, which is involved in myelotoxicity in mice [33]. Therefore, it is feasible that mice receiving 5-FU at pre-symptomatic stage will become pre-conditioned to better cope with the subsequent oxidative stress insult caused by SOD1G93A expression.

5-FU is a fluorinated analog of uracil. Both 5-FU and uracil use the same transport mechanism into cells. Uridine, a glycosylated uracil, is known to ameliorate the pathological phenotype of SOD1G93A mice [34]. As opposed to uridine experiment by Amante et al [34], we did not observe MN preservation in 5-FU treated mice. However, we cannot discard the possiblity of subtle changes in MN preservation or cell function. The spinal MN counts were carried out at 16 weeks of age, so we may have potentially missed alterations in MN numbers and reactive gliosis at earlier times. Therefore, further analysis is warranted on the effects of 5-FU in spinal cord neurons at earlier stages of the symptomatic phase.

Based on the presented transcript and protein data, there is no difference in the expression of SOD1G93A transgene in 5-FU vs. vehicle treated mice (S2 Fig). However, one of the most feasible possibilities is that 5-FU may interfere with SOD1 misfolding or aggregation. Misfolding can be transmitted between SOD1 molecules in cultured cells [35] and wild type SOD1 can be misfolded by expression of mutant FUS or TDP43 (TARDBP) [36], products of genes also affected in ALS. Besides these intermolecular actions, misfolding can be tranferred intercellularly between cells in culture [37]. Recently, 5-FU and 5-fluorouridine (5-Fur) has been shown to effectively block the spread of SOD1 misfolding in propagated misfolding cell culture assays [38]. Besides, 5-Fur has been shown to attenuate protein aggregation to the same extent as an antibody detecting misfolded SOD1 [39]. Therefore, although in vivo data on possible direct effects of 5-FU on SOD1 is missing, further evaluation of its potential effects on delaying disease propagation via antagonistic effect on misfolding and/or aggregation is warranted.

To conclude, the surprising finding that the anti-cancer drug 5-FU elicits protective effect on SOD1G93A model of ALS puts 5-FU and similar antimetabolites in the group of agents that could be possibly repurposed for the treatment of MN diseases. Studies focusing on target tissues and cell types, as well as resolving the molecular mechanisms of action of these substances on ALS models are needed.

## Supporting information

S1 Fig The effect of 5-FU administration on red blood cells, hemoglobin and hematocrit. Red blood cells (A), hemoglobin (B) and hematocrit (C) values are shown for pretreatment (0 days) and for 4, 14 and 20 days after 5-FU administration.
(TIF)

S2 Fig The effect of 5-FU administration on hSOD1-G93A transgene expression. Expresion of human SOD1 (hSOD1G93A) in mRNA (A) and protein (B) level in transgenic mice treated with 5-FU (SOD1 5-FU) and in vehicle controls (SOD1 Control).
(TIF)

S1 File Numerical data for the figures. (XLSX)
