# Supplementary figures and images for: Chemotherapeutic agent 5-fluorouracil increases survival of SOD1 mouse model of ALS
Source: PLoS One. 2019 Jan 14;14(1):e0210752. doi: 10.1371/journal.pone.0210752 (PMC6331125; doi:10.1371/journal.pone.0210752)

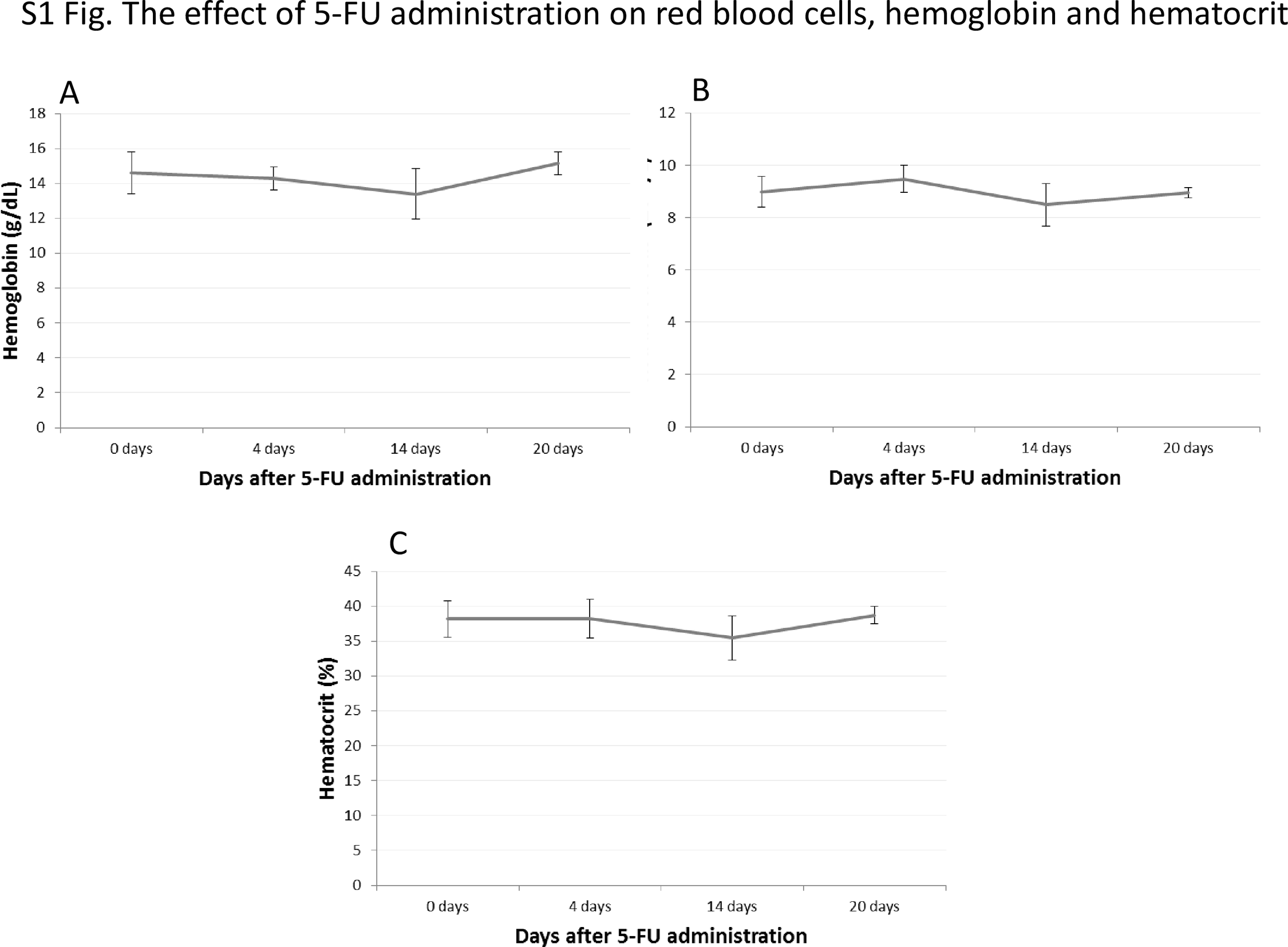

Supplement: S1 Fig — Red blood cells (A), hemoglobin (B) and hematocrit (C) values are shown for pretreatment (0 days) and for 4, 14 and 20 days after 5-FU administration. (TIF) [file pone.0210752.s001.tif]

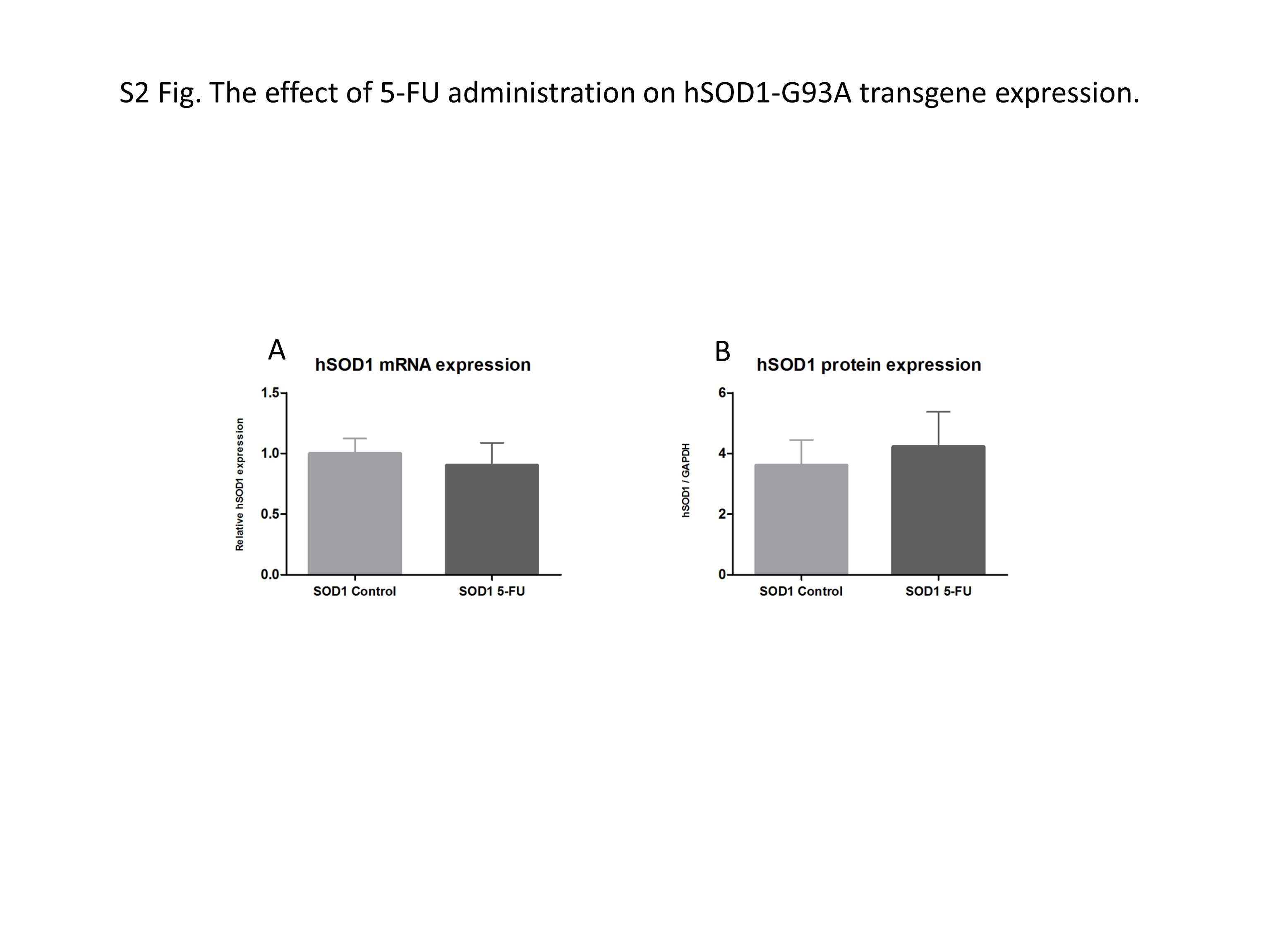

Supplement: S2 Fig — Expresion of human SOD1 (hSOD1G93A) in mRNA (A) and protein (B) level in transgenic mice treated with 5-FU (SOD1 5-FU) and in vehicle controls (SOD1 Control). (TIF) [file pone.0210752.s002.tif]
